# Cancer treatment and survivorship statistics, 2025: An urgent call to optimize health after cancer

**DOI:** 10.3322/caac.70017

**Published:** 2025-05-30

**Authors:** Lidia Schapira, Christine M. Duffy

**Affiliations:** ^1^ Department of Medicine Stanford University School of Medicine Palo Alto California USA; ^2^ Adult Cancer Survivorship Program Brown University Health Cancer Institute East Providence Rhode Island USA

**Keywords:** epidemiology, pediatric populations, survivorship, treatment

The publication of the American Cancer Society’s “Cancer Treatment and Survivorship Statistics, 2025” report affirms that the number of people living in the United States with a history of cancer is rising because of advances in detection and treatment that have improved survival.^1^, ^2^ In our opinion, it also presents a new opportunity to engage all stakeholders in the discourse on cancer survivorship. More cancers have become treatable and controllable, and the sheer number of survivors demands a concerted approach involving a trained health care workforce, accessible referral pathways, and adequate reimbursement for services rendered.

There are reasons to celebrate the findings as we learn that general cancer mortality continues to fall, with an overall incidence decline in men. Yet these improvements are not distributed equally among subpopulations because cancer mortality continues to rise in women, and we are presented with evidence of the persistence of disparities in access to life‐saving cancer treatment and receipt of guideline‐concordant care. For instance, there is evidence that patients with private insurance are twice as likely to receive recommended treatment for stage II–III colon cancer compared with patients who are uninsured, and Black patients are less likely than White patients to receive surgery for early stage colon and rectal cancers.^3^, ^4^ Disparities in receipt of guideline‐concordant care have been reported for patients with many solid tumors,^5^, ^6^ and this inevitably leads to worse outcomes.

The global disruption caused by the coronavirus disease 2019 pandemic will continue to be studied for years, but some of its consequential effects are beginning to surface. Among them are delays in screening and disruptions in care pathways that contribute to stage migration.^7^ In addition, the pandemic exposed fault lines across health care systems and exacerbations in disparities in cancer care. Other global events, including wars and famine that lead to massive migration, will undoubtedly have an impact on global cancer statistics in years to come.

Robust data banks are essential to advancing our understanding of long‐term outcomes in cancer survivors. Studies like the St Jude Lifetime Cohort and the Childhood Cancer Survivor Study have generated invaluable insights into survivorship in pediatric populations. The National Cancer Institute‐funded cancer epidemiology survivor cohorts, which were established to follow survivors over time to capture data on treatment exposures, long‐term health outcomes, and social determinants of health, are an important step that will inform future interventions and guidelines for care, but comprehensive population‐based surveillance of survivorship outcomes remains limited.^8^

Growing recognition of the toxicities and long‐term burdens associated with cancer treatments has driven efforts to de‐escalate therapy, aiming to balance efficacy with improved quality of life for cancer survivors. Major improvements in imaging techniques and genomic assays have made this possible for several cancers. Positron emission tomography‐computed tomography scans are now able to guide lymphoma treatment intensity and duration, thus allowing responders to benefit from abbreviated courses of cancer‐directed treatment. Genomic testing in early stage, hormonally sensitive breast cancer made it possible for women to avoid chemotherapy without compromising their survival. Sentinel node biopsies in breast cancer and melanoma have resulted in markedly lower rates of lymph node dissections, thus reducing the lifelong risk of lymphedema in these patient groups. Advances in surgical techniques in bladder and rectal cancers have allowed many more individuals to maintain functioning organs, significantly improving health‐related quality of life. Similarly, the shift toward watchful waiting in early stage prostate cancer for older men has spared many from experiencing known urinary and sexual complications of surgery.

Survivors of adolescent and young adult cancers, defined as individuals diagnosed with a primary malignancy between ages 15 and 39 years, constitute a population with unique developmental, psychosocial, and health‐related needs and are at risk of developing a range of chronic comorbidities. In fact, adolescent and young adult survivors generally experience two times the cumulative burden of severe to life‐threatening chronic health conditions compared with peers.^9^ To complicate matters, adolescent and young adult survivors are often treated in disparate settings (pediatric or adult) on dissimilar protocols that include different recommendations for longitudinal follow‐up. Specialized techniques and referral mechanisms are needed to ensure a seamless transition from acute cancer care to survivorship care, and this may include a transition from the pediatric setting to the adult setting, with each transition a risk for discontinuity and subsequent nonadherence to recommended screening guidelines.

The science and practice of survivorship care has flourished as a companion to the development of clinical therapeutics to address the long‐term consequences of cancer and its treatment. It includes a thorough and ongoing assessment of the patient with attention to surveillance and management of physical and psychosocial effects of cancer, prevention and surveillance of new cancer/recurring cancers, surveillance and management of chronic medical conditions, general health promotion and disease prevention, and care coordination.^10^ Cancer survivors may benefit from supportive services, including physical rehabilitation, and nutritional guidance and need access to specialty referrals in cardio‐oncology, oncofertility, pyscho‐oncology, endocrinology, lymphedema therapy, neurocognitive rehabilitation, pain management, sexual health, and more. Promoting tobacco cessation, exercise, and healthy weight and moderating alcohol consumption, which have been critical to reducing the cancer burden in the public, is essential in survivors given their shared risk factors.

What is neither clear nor standardized is who will care for cancer survivors. Models of care fall into broad categories and include specialist‐led care, shared care, primary care‐led care, and dedicated survivorship clinics, which offer multidisciplinary services. Advanced practice practitioners could play a key role in delivering survivorship services at cancer centers and clinics, and many have argued that this work is ideally suited for their scope of practice. Innovative practices are testing consultative models for survivorship care as well as fully integrating survivorship care into primary care. Customizing survivorship care based on diagnosis, exposures, as well as future risk (of recurrence or late effects from cancer therapies) allows for better utilization of resources. However, in the long run, most survivors will need to transition to generalist‐led care. Ensuring that the medical workforce is adequately prepared requires integrating survivorship education both during medical training and throughout continuing professional development ^11^, ^12^, ^13^, ^14^ Efforts to prepare cancer survivors by boosting self‐efficacy and self‐advocacy and arming them with concise treatment summaries and care plans have received considerable attention over the past 2 decades.^15^, ^16^ In fact, cancer survivors, communities, advocacy groups, and clinicians need more opportunities to co‐design models based on individual patient‐level characteristics and relevant outcome measures and must have evaluation mechanisms in place.^17^

What is clear is that we are not yet able to properly care for the 18.6 million cancer survivors in 2025 and that, without coordinated and strategic efforts, we will fall short of meeting the complex care needs of the estimated 26 million projected in the United States by 2030—a gap that poses a significant challenge to both oncology and primary care systems. In a fragmented health care system with survivorship expertise concentrated at major cancer centers and a shortage of primary care clinicians in rural and underserved areas, disparities in the quality of survivorship care and outcomes for cancer survivors are at risk of widening even further. We need innovative approaches that are grounded in high‐level evidence, where available, and interventions designed to disseminate best practices and patient‐facing interventions that are affordable and scalable.

The US national standards for survivorship care, published in 2024 by the National Cancer Institute, offer a clear blueprint for what cancer survivors and their families can expect after a cancer diagnosis.^18^ These standards were created to guide health care professionals and health systems in delivering comprehensive, personalized survivorship care that addresses the complex and evolving needs of survivors. They reflect significant advances in the understanding of survivorship but do not address the growing challenge of recognizing and treating toxicities from newer therapies like immunotherapy or drugs a patient may have received through enrollment in a clinical trial. One lesson we have learned is that a proactive approach to supportive care, particularly mental health services, can relieve the symptom burden both during and after the completion of curative‐intent treatment. Introducing supportive interventions earlier in the care trajectory can help survivors maintain better physical and emotional health over the long term, rather than waiting to address complications only after treatment ends. This approach aims to ensure that survivors are supported throughout their journey, improving overall quality of life and helping them transition more smoothly from active treatment to survivorship.

We face significant challenges in the years ahead as the number of cancer survivors continues to grow, the survivor population ages, novel treatments introduce new and often unpredictable toxicities, and workforce shortages persist among both oncologists and primary care physicians. To meet the growing needs of cancer survivors, we will need to innovate and explore new models of care. These models should be evaluated not only for their clinical effectiveness but also from the perspectives of survivors, caregivers, health care professionals, and society.

## CONFLICT OF INTEREST STATEMENT

Lidia Schapira is a consultant for Color Genomics and holds stock in Inculded Health. Christine M. Duffy disclosed no conflicts of interest.
